# Gallein potentiates isoniazid's ability to suppress *Mycobacterium tuberculosis* growth

**DOI:** 10.3389/fmicb.2024.1369763

**Published:** 2024-04-15

**Authors:** Ramesh Rijal, Richard H. Gomer

**Affiliations:** Gomer Lab, Department of Biology, Texas A&M University, College Station, TX, United States

**Keywords:** *Mycobacterium tuberculosis*, gallein, isoniazid, antibiotic tolerance, polyphosphate, metabolomics

## Abstract

*Mycobacterium tuberculosis* (*Mtb*), the bacterium that causes tuberculosis (TB), can be difficult to treat because of drug tolerance. Increased intracellular polyphosphate (polyP) in *Mtb* enhances tolerance to antibiotics, and capsular polyP in *Neisseria gonorrhoeae* potentiates resistance to antimicrobials. The mechanism by which bacteria utilize polyP to adapt to antimicrobial pressure is not known. In this study, we found that *Mtb* adapts to the TB frontline antibiotic isoniazid (INH) by enhancing the accumulation of cellular, extracellular, and cell surface polyP. Gallein, a broad-spectrum inhibitor of the polyphosphate kinase that synthesizes polyP, prevents this INH-induced increase in extracellular and cell surface polyP levels. Gallein and INH work synergistically to attenuate *Mtb*'s ability to grow in *in vitro* culture and within human macrophages. *Mtb* when exposed to INH, and in the presence of INH, gallein inhibits cell envelope formation in most but not all *Mtb* cells. Metabolomics indicated that INH or gallein have a modest impact on levels of *Mtb* metabolites, but when used in combination, they significantly reduce levels of metabolites involved in cell envelope synthesis and amino acid, carbohydrate, and nucleoside metabolism, revealing a synergistic effect. These data suggest that gallein represents a promising avenue to potentiate the treatment of TB.

## Introduction

Tuberculosis (TB) remains a significant global public health challenge, and in 2022, the causative bacterium *Mycobacterium tuberculosis* (*Mtb*) was responsible for ~1.6 million deaths worldwide (Bagcchi, 2023). During infection, *Mtb* encounters a variety of stressors originating from the host, and in response, employs adaptive physiological mechanisms to endure these stresses, promoting persistence and both tolerance to antibiotics and the development of drug tolerance (McCune, 1956; Deb et al., 2009; Jain et al., 2016; Liu et al., 2016; Mehta et al., 2016). These complexities not only demand prolonged treatment regimens but also contribute to the emergence of drug-resistant *Mtb* strains (Bagcchi, 2023). Notably, resistance to the primary antibiotic, isoniazid (INH), is a prevalent form of monoresistance in *Mtb*, which is associated with treatment failures and the emergence of multidrug-resistant TB (Bagcchi, 2023). *Mtb*'s resistance to the majority of antibiotics is attributed to the thickening of the cell envelope (Nguyen and Thompson, 2006; Silver, 2011), the activation of enzymes that modify antibiotics or their targets, and the action of efflux pumps (Jarlier and Nikaido, 1994; Nikaido, 1994). These mechanisms collectively reduce the efficacy of antibiotics.

Polyphosphate (polyP) is a chain of phosphate residues and is present in all kingdoms of life (Rao et al., 2009). PolyP metabolism has been linked to the virulence of pathogens such as *Mtb, Burkholderia mallei, Pseudomonas aeruginosa, Salmonella enterica*, and *Shigella flexneri* (Rashid et al., 2000; Kim et al., 2002; Tunpiboonsak et al., 2010; Chuang et al., 2013). A highly conserved bacterial enzyme, polyphosphate kinase (PPK), synthesizes polyP from ATP, while polyP levels are regulated by the action of exopolyphosphatase (PPX), an enzyme that removes terminal phosphate residues from a polyP chain (Kumble and Kornberg, 1995). The *Mtb* genome encodes two PPKs, PPK1 (Rv2984) and PPK2 (Rv3232c), as well as two PPXs, PPX1 (Rv0496) and PPX2 (Rv1026) (Cole et al., 1998).

Pathogenic bacteria lacking PPK or having reduced PPK levels exhibit defects in stress response, quorum sensing, growth, survival, and virulence (Rao et al., 1998; Rashid and Kornberg, 2000; Rashid et al., 2000; Chavez et al., 2004; Sureka et al., 2009; Zhang et al., 2010; Mookherjee et al., 2013; Manca et al., 2023). For instance, intracellular polyP is necessary for the survival of *Mtb* in host cells (Chavez et al., 2004; Sureka et al., 2007; Chuang et al., 2013; Singh et al., 2013), and deletion of PPK1 in *M. smegmatis* attenuates the survival of ingested *M. smegmatis* in human macrophages (Rijal et al., 2020). Conversely, increased intracellular polyP in *Mtb* causes increased tolerance to antibiotics (Thayil et al., 2011; Chuang et al., 2013, 2015; Singh et al., 2013). In addition to intracellular polyP, bacteria also have extracellular polyP. The pathogenic bacterium *Neisseria gonorrhoeae* has polyP in its capsule, and the polyP potentiates resistance to antimicrobials (Mookherjee et al., 2013; Manca et al., 2023). We observed that both *Mtb* and *M. smegmatis* accumulate extracellular polyP (Rijal et al., 2020). Treatment of *Mtb*-infected macrophages with a polyP-degrading recombinant exopolyphosphatase (ScPPX) reduced the *Mtb* burden in macrophages, suggesting that both intracellular and extracellular polyP potentiate the survival of *Mtb* in host cells (Rijal et al., 2020).

Given the absence of PPK enzymes in humans (Brown and Kornberg, 2004), bacterial PPKs could serve as potential targets for antituberculosis therapeutics. The small molecule gallein (Lillie et al., 1974) is a broad-spectrum PPK inhibitor (Neville et al., 2021; Roberge, 2021), and in this report, we find that gallein strongly potentiates the ability of INH to inhibit *Mtb* growth alone and in human macrophages.

## Results

### Gallein enhances the INH-mediated inhibition of *Mtb* growth

**Figure 1:**
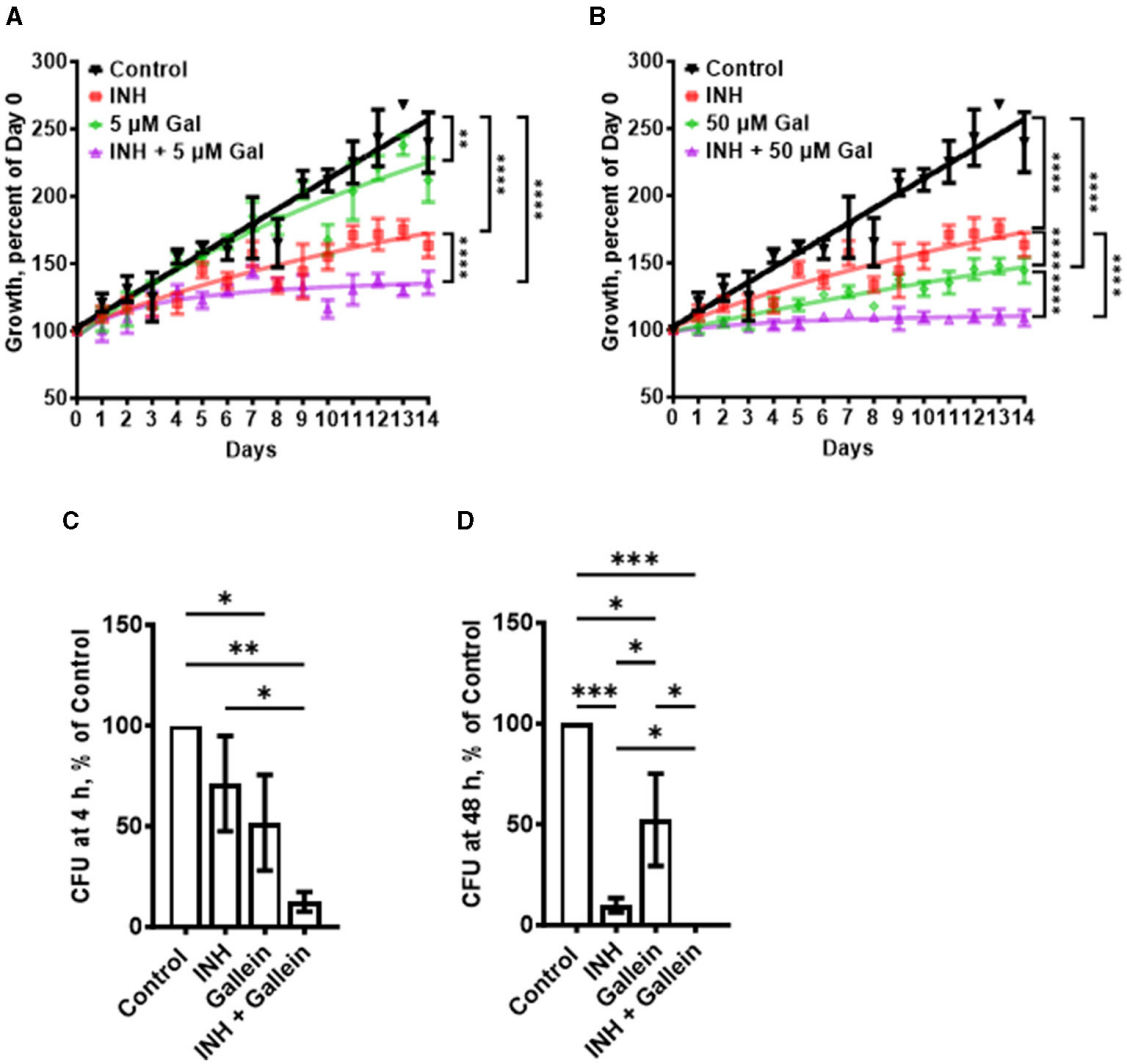
Gallein potentiates the ability of INH to inhibit *Mtb* growth both in *in vitro* culture and within macrophages. **(A, B)** *Mtb* cultures were grown for 14 days in the absence (Control) or presence of 1 μg/ml isoniazid (INH) and/or 5 μM **(A)** or 50 μM **(B)** gallein. The OD_600_ was measured daily, and growth was determined as a percentage of Day 0 OD_600_. **(C, D)** Viable ingested *Mtb* in macrophages, in the absence (Control) or presence of 1 μg/ml INH and/or 5 μM gallein, was determined as colony-forming units (CFU) at 4 h **(C)** and 48 h **(D)** after ingestion. CFU in the control was considered 100%. All values are mean ± SEM of three **(A, B)** and four (two females and two males) **(C, D)** independent experiments. *P < 0.05; **P < 0.01; ***P < 0.001, ****P < 0.0001 (two-way ANOVA with Dunnett's multiple comparisons test for **A, B**, and Mann-Whitney test for **C, D**).

PPK1 and PPK2, enzymes which synthesize polyP, are both necessary for *Mtb* viability (Sureka et al., 2009; Jagannathan et al., 2010). Ellagic acid derivatives from a medicinal plant inhibit PPK1 in *Pseudomonas aeruginosa* (Sarabhai et al., 2015). Gallein, a small molecule with similarity to ellagic acid, was identified as a potent inhibitor of both PPK1 and PPK2 in *P. aeruginosa* (Neville et al., 2021). Gallein also inhibits Gβγ subunit signaling in mammalian cells (Smrcka, 2013; Sanz et al., 2017). To determine if gallein affects *Mtb*, cells were cultured in the presence of different concentrations of gallein. Gallein at 0.005, 0.05, or 0.5 μM did not significantly affect *Mtb* growth, as assessed by an increase in OD_600_ values (Supplementary Figures S1A–C), while 5 μM gallein slightly slowed growth, and 50 μM gallein inhibited growth by ~80% (Figures 1A, B). Given that we observed complete inhibition of *Mtb* growth with 100 μg/ml INH in our experimental setup, we opted to utilize 1 μg/ml INH for all our assays. This choice aligns with reports from other research groups, which showed tolerance and resistance of *Mtb* to concentrations of INH >1 μg/ml (Ojha et al., 2008; Flentie et al., 2019). In the absence of gallein, 1 μg/ml INH caused a partial but not complete reduction in *Mtb* growth (Supplementary Figure S1D), and this effect was potentiated by 5 and 50 μM gallein (Figures 1A, B). The addition of 0.005, 0.05, or 0.5 μM gallein did not significantly enhance the effect of 1 μg/ml INH (Supplementary Figures S1A–C). To determine if the reduced growth with INH and/or 5 and 50 μM gallein causes a permanent effect on *Mtb*, the day 14 cultures were washed and resuspended in medium without INH or gallein and the growth was monitored (Supplementary Figure S1E). Surprisingly, exposure to INH caused *Mtb* cells to grow faster and exposure to gallein caused a slight reduction in growth. Together, these data indicate that a 14 day exposure of *Mtb* to 1 μg/ml INH and 5 or 50 μM gallein is bacteriostatic but not bactericidal. However, the apparent decrease in *Mtb* growth in the presence of INH and/or 5 or 50 μM gallein might also be attributed to changes cell wall size/shape.

In patients with tuberculosis, *Mtb* bacteria can be ingested by macrophages, and are able to survive inside the macrophages (Echeverría-Valencia, 2023). To determine if gallein affects the ability of *Mtb* to survive in macrophages, human macrophages, derived from circulating monocytes from healthy donors, were incubated with *Mtb* for 2 h, and the non-ingested *Mtb* were removed. The macrophages were then incubated with gallein and/or INH, and at 4 and 48 h after adding *Mtb* the macrophages were lysed with a detergent (0.1% Triton X-100) that does not kill *Mtb*, and the Supplementary Table S1). At both times, gallein decreased ingested *Mtb* viability compared to control, but the effects were similar at 4 and at 48 h (Figures 1C, D; Supplementary Table S1). One possibility for the stronger effect of gallein *in vitro* compared to the effect of gallein in macrophages is that compared to the concentration outside cells, the concentration of gallein inside a phagosome inside a macrophage is less, and in addition, the environment inside a phagosome is different from the environment in the *in vitro* experiment. In the presence of INH, gallein significantly decreased the viability of ingested *Mtb*, with no detected surviving bacteria at 48 h (Figures 1C, D; Supplementary Table S1). Together, these results suggest that for both free *Mtb* and *Mtb* in macrophages, gallein inhibits growth and enhances INH's ability to inhibit growth.

### Isoniazid increases the accumulation of polyp

**Figure 2:**
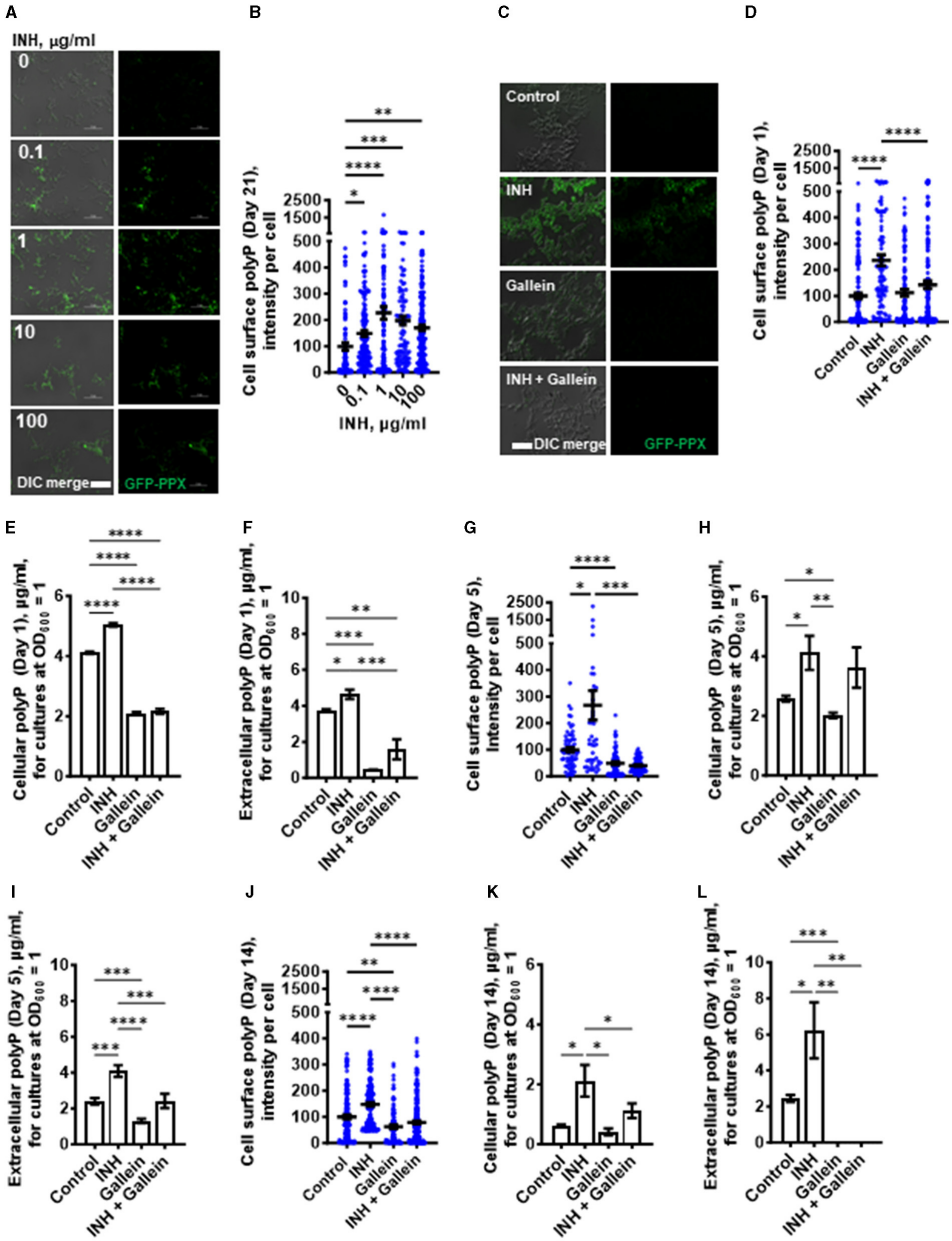
INH induces accumulation of *Mtb* cell surface, cellular, and extracellular polyP. **(A)** *Mtb* were cultured for 21 days in the presence of the indicated concentrations of INH and then stained with GFP-PPX. Differential interference contrast (DIC) merged with fluorescence are at the left, and fluorescence images are at the right. Representative images from at least three independent experiments are shown. Bars are 10 μm. **(B)** GFP-PPX staining intensity per cell, as a measure of *Mtb* cell surface polyP per cell, was determined from **(A)**. Black bars are means. In **(B, D, G, J)**, the staining intensities are in arbitrary units with the control averages set to 100. **(C)** DIC merge and fluorescence images of *Mtb* grown for 1 day in the absence or presence of 1 μg/ml INH and/or 5 μM gallein and stained with GFP-PPX are displayed. Representative images from at least three independent experiments are shown. Bar is 10 μm. **(D)** GFP-PPX staining intensity per cell, as a measure of *Mtb* cell surface polyP per cell, was determined from **(C)**. Black bars are means. **(E)** *Mtb* were grown for 1 day in the absence or presence of 1 μg/ml INH and/or 5 μM gallein. The OD_600_ was measured, cells were lysed, and cellular polyP levels for cultures at OD_600_ = 1 were determined. **(F)** *Mtb* were grown for 1 day in the absence or presence of 1 μg/ml INH and/or 5 μM gallein. The OD_600_ was measured, and supernatant conditioned medium was collected. Extracellular polyP levels for cultures at OD_600_ = 1 were determined. **(G–L)** *Mtb* were grown for 5 days **(G–I)** or 14 days **(J–L)** in the absence or presence of 1 μg/ml INH and/or 5 μM gallein. Levels of cell surface polyP **(G, J)**, cellular polyP **(H, K)**, and extracellular polyP **(I, L)** were determined as described in **(C–F)**. All values are mean ± SEM of three independent experiments. *P < 0.05; **P < 0.01; ***P < 0.001, ****P < 0.0001 (one-way ANOVA with Tukey's multiple comparisons test).

Increased intracellular polyP in *Mtb* causes increased tolerance to antibiotics (Thayil et al., 2011; Chuang et al., 2013, 2015; Singh et al., 2013). To determine if exposure of *Mtb* to INH potentiates accumulation of polyP (Thayil et al., 2011; Chuang et al., 2015), *Mtb* cells were cultured in the presence of INH. After exposure to INH for 21 days, the *Mtb* cells were fixed without permeabilizing the cells and then stained with the GFP-tagged polyP binding domain of *Escherichia coli* PPX (GFP-PPX) (Xie et al., 2019). For unknown reasons, the cells showed a wide range of staining intensities (Figures 2A, B). Although INH concentrations from 0.1 to 100 μg/ml increased the average amount of cell-surface polyP, 1 μg/ml INH had a stronger effect in increasing the amount of cell surface polyP (Figures 2A, B), which aligns with our previous decision to use 1 μg/ml INH for all of our assays. The apparently weaker effect of 10 and 100 μg/ml INH may be due to the higher concentrations of INH causing a general disruption in cell metabolism, including the ability to increase cell surface polyP. A 1 day exposure of *Mtb* to 1 μg/ml INH also increased cell-surface and total cellular polyP (Figures 2C–E). We previously observed that *Mtb* cells accumulate extracellular polyP (Rijal et al., 2020), and at 1 day INH also increased the accumulation of extracellular polyP (Figure 2F). At 5 and 14 days, 1 μg/ml INH significantly increased cell-surface, total cellular, and extracellular polyP (Figures 2G–L). At 14 days, *Mtb* controls had, using an arbitrary cutoff, 23% of cells with low levels of cell surface polyP, cells treated with gallein in the presence or absence of INH had 43% of cells with low levels of cell surface polyP, but none of the cells treated with INH had low levels of cell surface polyP (Supplementary Figure S2A). Together, these findings suggest that INH induces the accumulation of cellular, extracellular, and cell surface polyP.

### Gallein prevents the INH-induced accumulation of extracellular and cell surface polyP

One possible mechanism for the effect of gallein on *Mtb* growth is that gallein, by inhibiting PPKs, decreases polyP levels. We measured cell surface, cellular and extracellular polyP levels in *Mtb* in the absence or presence of 1 μg/ml INH and/or 5 μM gallein. At 1 day, in the absence of INH, gallein did not decrease cell surface polyP, but did block the INH-induced increase in cell surface polyP, bringing the cell surface polyP levels to levels comparable to control cells (Figure 2D). Gallein decreased cellular polyP in both the absence and presence of INH (Figure 2E). Gallein also decreased extracellular polyP in both the absence and presence of INH (Figure 2F). At day 1, INH and gallein did not significantly affect levels of *Mtb ppk1* and *ppk2* mRNAs (Supplementary Figure S3A), but at day 5, INH and gallein significantly reduced the levels of *Mtb ppk2* mRNAs (Supplementary Figure S3B), suggesting that the effects on polyP levels are mediated by changes in the levels of *ppk2* mRNAs. At 5 days, gallein significantly decreased cell surface polyP in both the absence or in the presence of INH (Figure 2G). At 5 days, gallein decreased cellular polyP in the absence of INH (Figure 2H). Gallein also decreased extracellular polyP in both the absence and presence of INH (Figure 2I). In the presence of INH, gallein decreased extracellular polyP to levels comparable to control cells (Figure 2I). At 14 days, gallein significantly decreased cell surface polyP in both the absence or in the presence of INH (Figure 2J). At 14 days, gallein did not significantly decrease cellular polyP in the absence of INH (Figure 2K), but significantly decreased cellular polyP in the presence of INH (Figure 2K). Gallein decreased extracellular polyP in both the absence and presence of INH (Figure 2L). At 14 days, in the absence or presence of INH, gallein decreased extracellular polyP to an undetectable level (Figure 2L). At 14 days, in the presence of the combination of INH and gallein, cellular debris was visible, indicating cell death (Supplementary Figure S2B). For unknown reasons, in control cells, cellular and extracellular polyP levels tended to decrease with the age of the cultures. Together, these results suggest that INH increases total, cell surface, and extracellular polyP, and that gallein blocks this effect except for total polyP at day 5.

### Gallein prevents INH-induced thickening of the *Mtb* cell envelope

**Figure 3:**
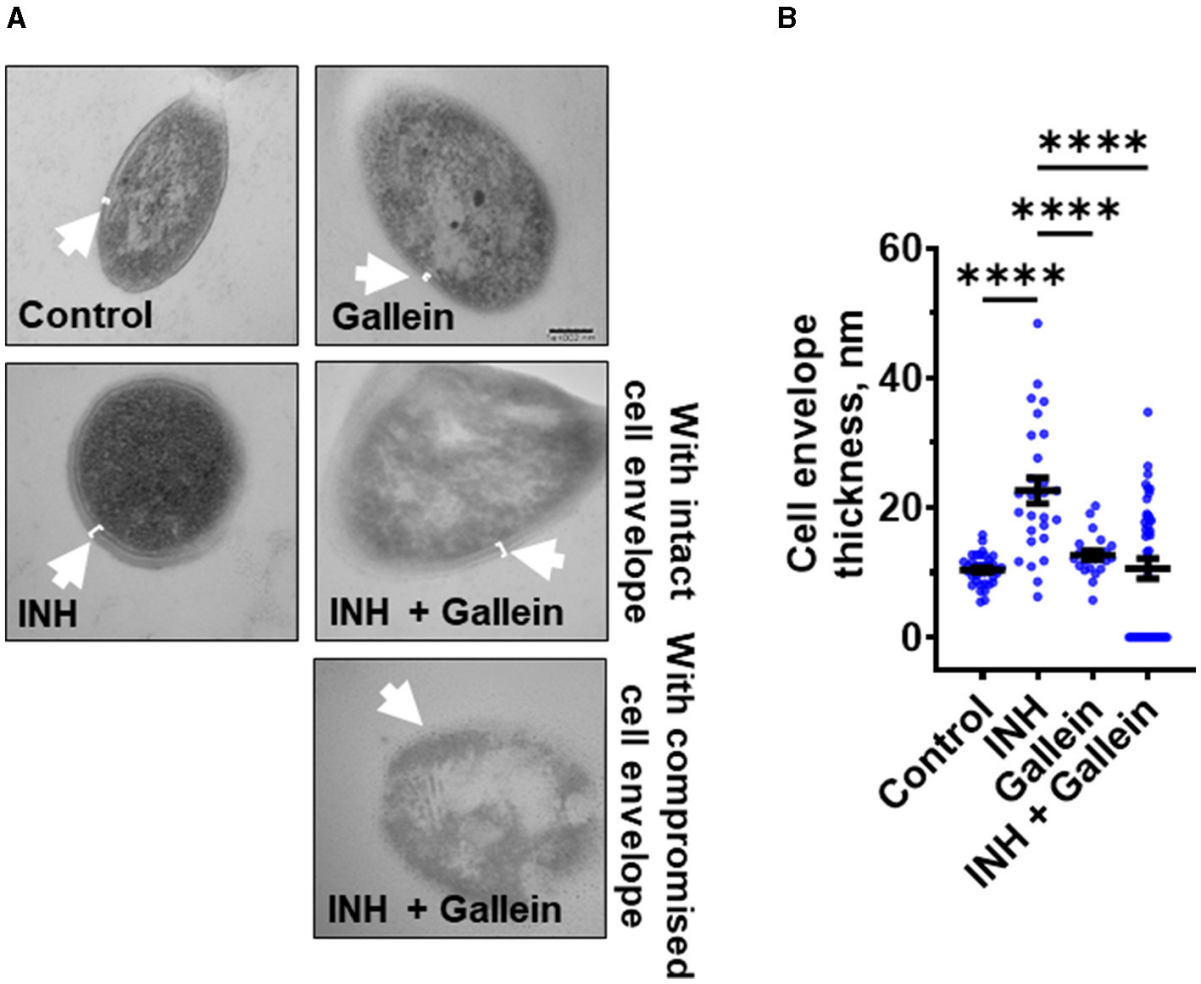
Gallein inhibits INH-induced *Mtb* cell envelope thickening. **(A)** Transmission electron microscopy images of *Mtb* treated without or with 1 μg/ml isoniazid (INH) and/or 5 μM gallein for 14 days. Representative images are from at least three independent experiments. The arrows indicate the cell envelope. Bar is 100 nm. **(B)** Quantification of cell envelope thickness. Values are mean ± SEM of at least 25 cells from three independent experiments. ****P < 0.0001 (one-way ANOVA with Tukey's multiple comparisons test).

When exposed to INH, *Mtb* undergoes cell envelope thickening and reduces cell envelope permeability as an adaptive response to withstand INH (Chuang et al., 2015). To investigate whether gallein can reverse the effects of INH on cell envelope morphology, *Mtb* was treated with 1 μg/ml INH and/or 5 μM gallein for 14 days, and the *Mtb* cell wall was visualized using transmission electron microscopy (TEM). As previously observed (Chuang et al., 2015), INH increased *Mtb* cell envelope thickness (Figures 3A, B). Gallein alone did not significantly affect cell envelope thickness. For cells exposed to both INH and gallein, the average cell envelope thickness was comparable to control cells, but there were two distinct populations of *Mtb* cells (Figures 3A, B). For 43.0 ± 2.6% (mean ± SEM, *n* = 3) of the *Mtb* in the presence of INH and gallein, there was a detectable cell envelope, while the remaining *Mtb* had no detectable cell envelope (Figures 3A, B). These results suggest that in the absence of INH, gallein does not inhibit growth by affecting cell envelope thickness, and that the combination of INH and gallein causes the formation of two populations of cells, one with a detectable cell envelope (which for many of these cells is thicker than that of control cells), and one with a compromised cell envelope.

### Gallein and INH work synergistically to downregulate key metabolic pathways

To evaluate the impact of gallein on *Mtb* metabolism, *Mtb* were exposed to 1 μg/ml INH and/or 5 μM gallein for 24 h, then incubated with a viability dye for 12 h, and the resulting fluorescence signal was measured. The conversion of the non-fluorescent dye resazurin to the fluorescent resorufin product serves as a measure of metabolism (Cho et al., 2015; Lescat et al., 2019), and is also used to assess the susceptibility of *Mtb* to antimicrobial compounds (Parish and Stoker, 1998). In comparison to the control, *Mtb* exposed to INH showed increased metabolic activity (Supplementary Figure S4A). Gallein decreased metabolic activity in the presence or absence of INH (Supplementary Figure S4A).

**Figure 4:**
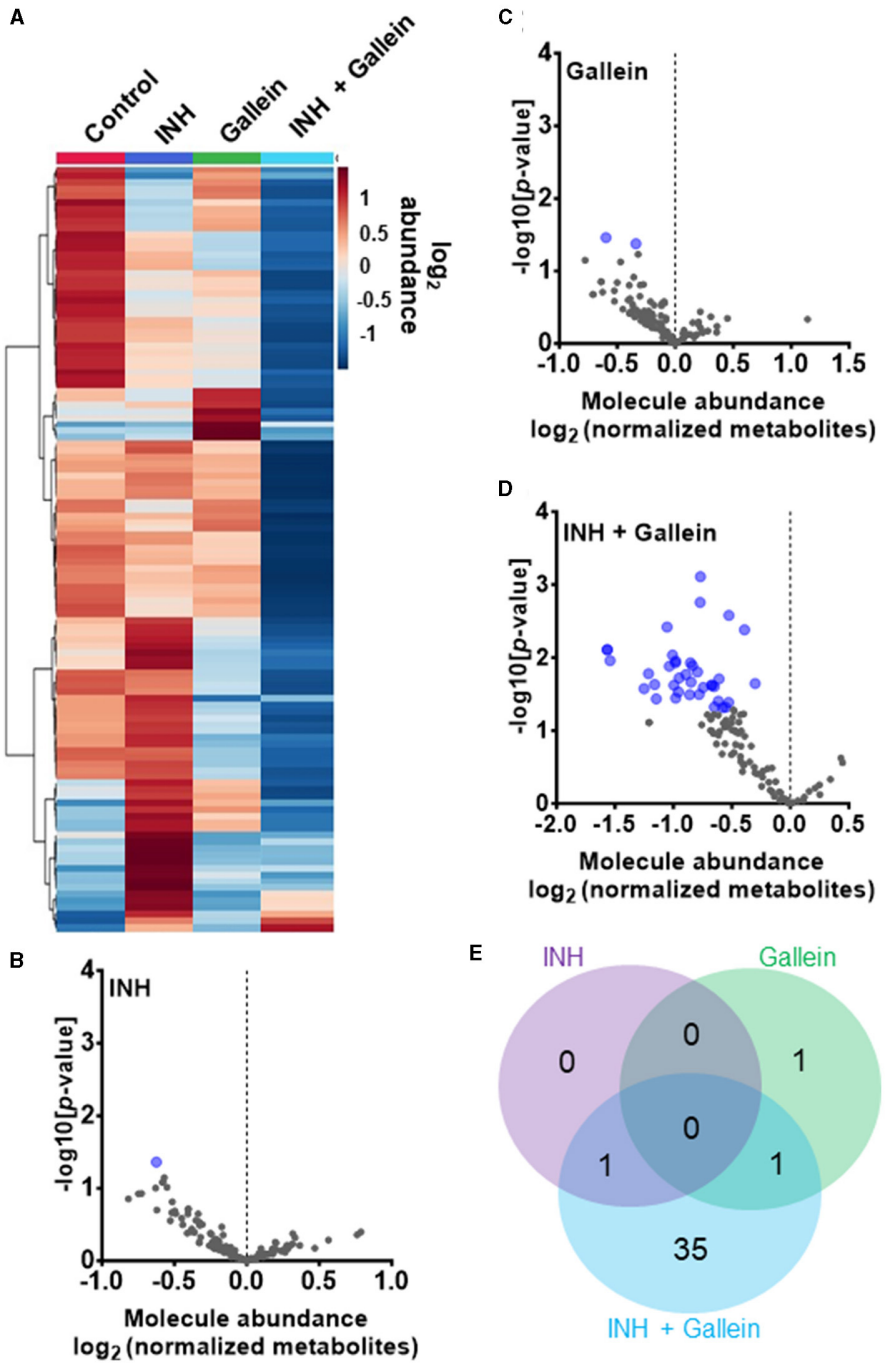
INH and gallein work synergistically to reduce metabolite levels. **(A)** Hierarchical clustering heatmap of 119 metabolites from *Mtb* treated with or without 1 μg/ml INH and/or 5 μM gallein for 24 h. Rows represent the mean abundance of individual metabolites detected in all three independent experiments, normalized to total protein content. The data were generated using the metabolomics data analysis tool MetaboAnalyst (https://www.metaboanalyst.ca/), and values are depicted on a log2 scale. **(B–D)** The abundance of individual metabolites detected in all three independent experiments was normalized to total protein content, and the mean fold change of normalized abundance of individual metabolites relative to the control on a log2 scale was plotted against the –log10 (*p*-value) to generate volcano plots. Blue dots indicate metabolites with relative levels having *P* < 0.05 (Student's *t*-test). **(E)** A Venn diagram depicts unique and common metabolites that were significantly reduced in **(B–D)**. All values represent the mean ± SEM of three independent experiments.

Three clinically used tuberculosis drugs, namely INH, rifampicin (RIF), and streptomycin (STREP), induce a common pattern of metabolic alterations (Nandakumar et al., 2014). To determine how gallein affects metabolites, *Mtb* was exposed to 1 μg/ml INH and/or 5 μM gallein for 24 h. *Mtb* metabolites were extracted and subjected to untargeted metabolomics analysis, which identified a total of 119 metabolites. Partial least-squares discriminant analysis (PLS-DA) revealed a clear distinction between the *Mtb* populations treated with gallein and INH in comparison to the control. Component 2, responsible for the largest proportion of the total variance in metabolites (8.6%), placed the gallein and INH treated samples considerably apart from the control, gallein, or INH treated samples (Supplementary Figure S4B). This observation suggests that the metabolic changes induced by gallein and INH were more substantial than those caused by gallein or INH alone (Supplementary Figure S4B). A heatmap also indicated that although INH alone and gallein alone have some effect on metabolites, the combination of gallein and INH has a more profound effect (Figure 4A).

As previously observed using a variety of INH concentrations (Rohde and Sorci, 2020), 1 μg/ml INH significantly reduced the levels of nicotinamide adenine dinucleotide (NAD^+^) (Figure 4B; Table 1). Other workers also found that 6.4 μg/ml INH alters levels of many metabolites (Nandakumar et al., 2014). We observed that gallein significantly reduced the levels of deoxythymidine diphosphate (dTDP) and biotin (Figure 4C; Table 1). The combination of gallein and INH significantly reduced the levels of 37 metabolites (Figure 4D; Table 1) including NAD^+^ and dTDP but not biotin (Figure 4E). These pathways are detailed in Table 2 and include nucleoside and nucleotide biosynthesis and degradation, carrier, cofactor, and vitamin biosynthesis, amino acid biosynthesis and degradation, carbohydrate biosynthesis and degradation, aminoacyl tRNA charging, cell structure biosynthesis, metabolic regulator biosynthesis, fermentation, and fatty acid and lipid degradation pathways. Gallein, INH, or the combination of gallein and INH, did not significantly upregulate any detectable metabolite. These data indicate that gallein and INH work synergistically to impact the majority of key metabolic pathways, contributing to the inhibition of *Mtb* growth.

**Table 1:** Metabolites significantly altered in *Mtb* treated with INH and/or gallein, as compared to the control.

**Conditions**	**Metabolites**	**GO terms**	**Pathways**
INH	NAD+	GO:0019674—NAD metabolic process	NAD metabolism
Gallein	Deoxythymidine diphosphate (dTDP)	GO:0046072—dTDP metabolic process	dTDP-sugar biosynthesis
	Biotin	GO:0006768—Biotin metabolic process	Biotin biosynthesis
INH + Gallein	dTMP	GO:0046073—dTMP metabolic process	Pyrimidine deoxyribonucleosides salvage
	Coenzyme A	GO:0015936—Coenzyme A metabolic process	Coenzyme A biosynthesis
	Guanosine	GO:0042453—Deoxyguanosine metabolic process	Guanine and guanosine salvage II
	2-deoxy-D-ribose 5-phosphate	GO:0019692—Deoxyribose phosphate metabolic process	2′-deoxy-α-D-ribose 1-phosphate degradation
	UDP-α-D-glucose	GO:0006011—UDP-glucose metabolic process	Trehalose biosynthesis I
	Glycerol 2-phosphate	GO:0006072—Glycerol-3-phosphate metabolic process	Cytidine-5′-diphosphate-glycerol biosynthesis
	NAD+	GO:0019674—NAD metabolic process	NAD Metabolism
	dCDP	GO:0046087—Cytidine metabolic process	CDP-sugar biosynthesis
	Nicotinate	Pyridine carboxylic acid	NAD biosynthesis
	5′-deoxyadenosine	GO:0009119—Ribonucleoside metabolic process	5′-Deoxyadenosine degradation
	L-methionine	GO:0006555—Methionine metabolic process	L-methionine biosynthesis
	4-aminobutanoate	GO:0009448—Gamma-aminobutyric acid metabolic process	4-Aminobutanoate degradation
	Adenylosuccinate	N6-(1,2-dicarboxyethyl)AMP adenylosuccinic acid	Adenosine ribonucleotides *de novo* biosynthesis
	*N*-acetyl-L-glutamate 5-semialdehyde	*N*-acetyl-L-glutamate semialdehyde	L-arginine biosynthesis II (acetyl cycle)
	L-citrulline	GO:0000052—Citrulline metabolic process	L-citrulline biosynthesis
	L-lysine	GO:0006553—Lysine metabolic process	L-lysine biosynthesis
	2′-deoxyinosine	2′-deoxyinosine	Superpathway of purine deoxyribonucleosides degradation
	GDP-α-D-mannose	GO:0019673—GDP-mannose metabolic process	Phosphatidylinositol mannoside biosynthesis
	ADP	GO:0046031—ADP metabolic process	Glycolysis I (from glucose 6-phosphate)
	Pipecolate	Pipecolate	L-lysine degradation II (L-pipecolate pathway)
	dTDP	GO:0046072—dTDP metabolic process	dTDP-sugar biosynthesis
	2-hydroxy-5-oxoproline	2-hydroxy-5-oxoproline	2-hydroxy-5-oxoproline
	NADPH	GO:0006739—NADP metabolic process	NAD(P)/NADPH interconversion
	5-oxo-L-proline	5-oxo-L-proline metabolism	5-oxo-L-proline metabolism
	N-acetyl-L-glutamine	N-acetyl-L-glutamine	*N*-acetyl-L-glutamine
	dAMP	GO:0046033—AMP metabolic process	Purine deoxyribonucleosides salvage
	Methylmalonate semialdehyde	2-methyl-3-oxopropanoate	Methylmalonate semialdehyde
	An *N*-acyl-L-aspartate	*N*-acyl-L-aspartate	An *N*-acyl-L-aspartate
	NADP+	GO:0006739—NADP metabolic process	NAD phosphorylation and dephosphorylation
	Adenosine 3′,5′-bisphosphate	GO:0046031—ADP metabolic process	Mycobacterial sulfolipid biosynthesis
	2′-deoxycytidine	GO:0047844—Deoxycytidine deaminase activity	Superpathway of pyrimidine deoxyribonucleosides degradation
	Trehalose	GO:0005991—Trehalose metabolic process	Trehalose biosynthesis
	L-proline	GO:0006560—Proline metabolic process	L-proline degradation
	D-ribose 5-phosphate	GO:0043456—Regulation of pentose-phosphate shunt	Pentose phosphate pathway (non-oxidative branch) I
	UMP	GO:0046049—UMP metabolic process	UMP biosynthesis
	Pyridoxine	GO:0008614—Pyridoxine metabolic process	Vitamin B6 degradation I

Metabolites were assigned Gene Ontology (GO) terms and classified into metabolic pathways.

**Table 2:** Characterization of metabolites significantly altered in *Mtb* treated with the combination of INH and gallein compared to control.

**Metabolites**	**Pathways**
UMP dCDP dTMP dTDP Guanosine Adenosine Adenylosuccinate ADP	Nucleoside and nucleotide biosynthesis
NADP^+^ NAD^+^ NADPH L-methionine Nicotinate	Carrier, cofactor, and vitamin biosynthesis
L-proline L-citrulline L-methionine L-lysine *N*-acetyl-L-glutamate 5-seminaldehyde	Amino acid biosynthesis
UDP-α-D-glucose GDP-α-D-glucose D-ribose 5-phosphate Trehalose	Carbohydrate biosynthesis
Methionine L-lysine L-proline	Aminoacyl tRNA charging
D-ribose 5-phosphate UDP-α-D-glucose	Cell structure biosynthesis
UDP-α-D-glucose Trehalose	Metabolic regulator biosynthesis
5-oxo-L-proline D-ribose 5-phosphate	Fermentation
2-deoxy-D-ribose 5-phosphate Adenosine Guanosine D-ribose 5-phosphate	Nucleoside and nucleotide degradation
UDP-α-D-glucose Trehalose Glycerol	Carbohydrate degradation
L-citrulline Methionine 4-aminobutanoate	Amino acid degradation
Glycerol Coenzyme A	Fatty acid and lipid degradation

Metabolites were grouped based on their involvement in metabolic pathways.

## Discussion

**Figure 5:**
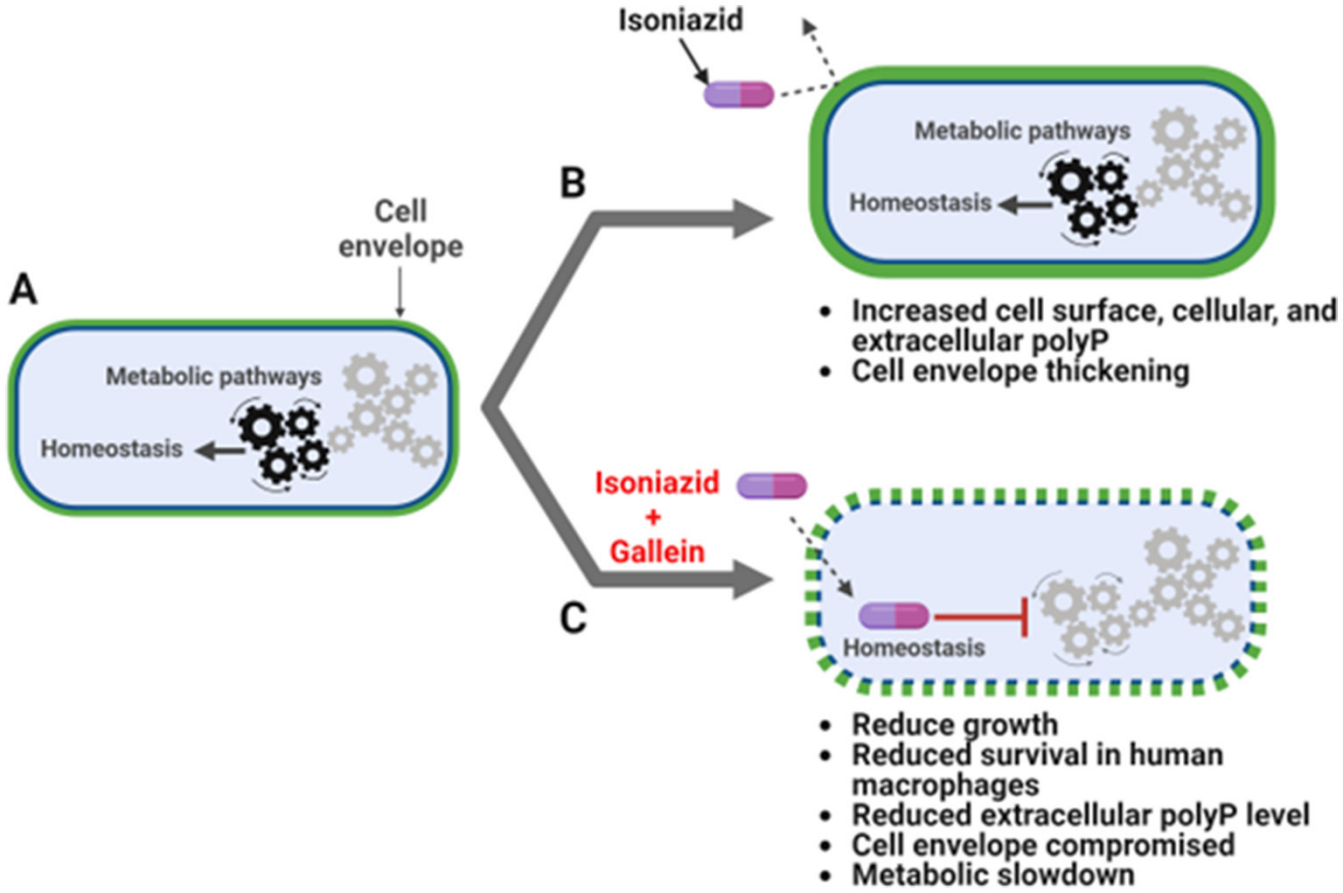
INH and gallein synergistically alter cellular homeostasis. **(A)** *Mtb* under no antibiotic pressure maintain homeostasis. **(B)** Under isoniazid (INH) pressure, *Mtb* grow slowly in *in vitro* culture, and increase the accumulation of cell surface, cellular, and extracellular polyP, as well as thickness of the cell envelope. **(C)** When exposed to both INH and gallein, *Mtb* exhibit decreased metabolic activity, decreased cell surface and extracellular polyP, and decreased cell envelope formation and integrity in many cells, resulting in very slow growth in both *in vitro* culture and within macrophages. The pathway diagram was created using BioRender.com.

Bacteria resist antibiotics by activating drug efflux pumps and/or enzymes which modify the antibiotic or its target (Nguyen and Thompson, 2006). Cellular metabolic rearrangements can also cause antibiotic resistance (Morris et al., 2005; Smith and Romesberg, 2007; Allison et al., 2011; Baek et al., 2011; Nguyen et al., 2011; Nandakumar et al., 2014). In this report, we show that the antibiotic INH causes *Mtb* to increase the accumulation of cell surface, cellular, and extracellular polyP. Gallein, a bacterial PPK 1 and 2 inhibitor, inhibits *Mtb* cell surface, cellular, and extracellular polyP accumulation in both the presence and absence of INH, and in the presence of INH inhibits *Mtb* cell envelope formation in some but not all *Mtb* cells. Both INH and gallein have modest effects on metabolite levels, but the combination of INH and gallein strongly reduces levels of metabolites in several metabolic pathways. Possibly as a consequence of the effects of gallein on polyP levels, cell envelope formation, and metabolites, gallein inhibits *Mtb* growth in both *in vitro* culture and in human macrophages, and strongly potentiates INH inhibition of *Mtb* growth in *in vitro* culture and in human macrophages (Figure 5).

We observed a significant increase in cellular, extracellular, and cell surface polyP in response to INH. Compared to log-phase cells, stationary phase *Mtb* have more polyP and are more resistant to killing by INH (Herbert et al., 1996; Rijal et al., 2020), and capsular polyP accumulation protects *N. gonorrhoeae* from antibiotics (Mookherjee et al., 2013; Manca et al., 2023). Combining these observations, one possibility is that *Mtb* increase polyP in response to INH as a protective measure. The antibiotic rifampicin causes *Mtb* to thicken its capsular outer layer and increase the net negative charge of the cell surface to reduce rifampicin permeability (Sebastian et al., 2020). It is possible that the INH-induced increase in the accumulation of the highly negatively charged polyP on the cell surface might similarly increase the net negative charge of the cell surface, reducing permeability to INH.

At 5 μM, gallein inhibits extracellular polyP levels in *Mtb* and inhibits INH-induced increases in cell surface and extracellular polyP. Gallein treatment of *P. aeruginosa* required concentrations exceeding 25 μM to reduce intracellular polyP accumulation, and 100 μM to mimic the effects of PPK deletion (Neville et al., 2021). Our findings suggest that gallein exhibits a greater effectiveness on *Mtb* compared to *P. aeruginosa*.

*Mtb* can differentiate into heterogeneous populations, such as non-replicating persisters and growing bacteria with the capacity to become persisters (Zhang, 2007). *Mtb* treated with INH causes a rapid killing of cells followed by a reduction in the killing rate as the number of persister cells increases (Van den Bergh et al., 2017; Vilchèze and Jacobs, 2019). In *Clostridiodes difficile*, a gram-positive bacteria, some cells in a population stochastically become antibiotic tolerant persisters (Álvarez et al., 2018). *C. difficile* forms metabolically dormant spores that resist antibiotic pressure and persist in the host (Deakin et al., 2012). Two distinct morphotypes of *C. difficile* spores exist, one with thick outer surface layer and the other with thin outer surface layer (Pizarro-Guajardo et al., 2016a,b). Multi-drug resistant *Mtb* have thicker cell envelopes (Schami et al., 2023). INH caused many, but not all, *Mtb* cells to have thickened cell envelopes. In response to INH and gallein, *Mtb* formed two distinct populations of cells: one with cell envelope thicknesses comparable to INH-treated cells, and the other with no discernible cell envelope. One possibility is that under combined INH and gallein pressure, either stochastic differentiation or asymmetric cell division gives rise to ~43% of cells with an intact cell envelope that have the potential to become slow growing persisters, and ~57% of cells with compromised cell envelope that eventually die, and this might be the reason we observed no net increase in *Mtb* cell growth in the presence of 50 μM gallein and INH.

For control *Mtb* cells, there was a large variation in cell surface polyP levels, and INH increased both the average cell surface polyP levels and cell envelope thickness. At day 14, although there was a clear bimodal distribution of cell envelope thicknesses in the presence of INH and gallein, there was no observable bimodal distribution of cell-surface polyP levels. This suggests that there is not a strict correlation between cell-surface polyP level and cell envelope thickness, and that the effect of combined INH and gallein on cell wall thicknesses may be due to effects on additional pathways in addition to cell surface polyP.

While 5 μM gallein enhances INH's ability to inhibit *Mtb* growth in both *in vitro* culture and within macrophages, the extracellular and cell surface polyP levels from cells treated with either gallein alone or the combination of gallein and INH were similar. This implies that gallein, by reducing polyP levels, may lead to *Mtb* envelope destabilization, rendering it susceptible to INH in both *in vitro* culture and human macrophages. In addition, the effect of gallein and/or gallein with INH on cell viability does not correlate with the effects of these compounds on cellular, extracellular, or cell-surface polyP accumulation. Although polyP levels seemed comparable in cells treated with gallein alone or in combination with INH, the synergistic action of gallein and INH may influence additional pathways to curb *Mtb* growth.

An INH-NAD adduct inhibits Enoyl acyl carrier protein reductase (InhA), a key enzyme involved in mycolic acid biosynthesis in *Mtb* (Quemard et al., 1995; Rawat et al., 2003). *Mtb* residing in macrophages have been shown to increase levels of NADH to prevent INH-mediated inhibition of InhA (Bhat et al., 2016). It is possible that the increase in NADH levels in response to INH may have led to the conversion of the non-fluorescent blue dye resazurin into the fluorescent pink product resorufin (Rampersad, 2012), which may not necessarily indicate increased metabolic activity in *Mtb* treated with INH.

*Mtb* uses the glucose disaccharide trehalose as a core component of cell surface glycolipids important for virulence, and during the transition into a non-replicating persister phase, it metabolizes trehalose to generate ATP (Elbein et al., 2003; Kalscheuer and Koliwer-Brandl, 2014; Koliwer-Brandl et al., 2016). Deletion of PPK1 in *Mtb* perturbs key metabolites involved in trehalose metabolism (Chugh et al., 2024). Although gallein alone did not significantly alter trehalose levels, the combination of INH and gallein reduced the levels of trehalose and components necessary for trehalose biosynthesis, such as UDP-α D-glucose (De Smet et al., 2000), and mimics effects of PPK1 deletion (Chugh et al., 2024), suggesting that the combination of INH and gallein compromises trehalose-dependent cell-surface glycolipid, and thus cell envelope, formation as we observed under TEM, and by decreasing trehalose levels, reduces this source of energy to prevent persister formation.

Loss of PPK1 in *Mtb* reduces levels of glucose 6-phosphate, the pentose phosphate pathway component ribose-5-phosphate, and tricarboxylic acid cycle components such as citric acid, succinate, fumarate, and malate (Chugh et al., 2024). These components of central carbon metabolism are important for amino acid biosynthesis (Umbarger, 1978; Chugh et al., 2024). Reduced levels of metabolites from carbohydrate and amino acid biosynthesis pathways in *Mtb* treated with the combination of INH and gallein suggests that these perturbation in central carbon and amino acid metabolism mimics PPK1 loss in *Mtb* (Chugh et al., 2024).

Increased polyP accumulation in *Mtb* increases levels of pyrophosphate, NAD^+^, NADH, nicotinamide, malate, succinate, 2-methyl citrate, acetyl-CoA, and metabolites belonging to arginine metabolism such as arginine, citrulline, and ornithine (Chuang et al., 2016; Chugh et al., 2024). Reduced levels of NAD^+^ and nicotinate from carrier, cofactor, and vitamin biosynthesis in *Mtb* treated with INH alone or the combination of INH and gallein indicate that INH alone or combination of INH and gallein perturbs polyP accumulation. Since few metabolites from carrier, cofactor, and vitamin biosynthesis pathway were altered compared to *Mtb* with increased polyP accumulation as a result of loss of PPX, it is also possible that INH and gallein affect some components of the pathway that do not involve the polyP pathway. Increased polyP accumulation reduces the nucleoside and nucleotide biosynthesis pathway components such as guanosine (Chugh et al., 2024), which suggests that reduced nucleoside and nucleotide biosynthesis pathway components in *Mtb* treated with the combination of INH and gallein might also be independent of the polyP pathway.

Gallein significantly reduced the levels of biotin. *Mtb* relies on biotin synthesis for its survival during infection, and the disruption of the biotin biosynthesis pathway results in cell death rather than growth arrest (Dey et al., 2010). This suggests that gallein may inhibit *Mtb* growth by promoting cell death rather than merely inhibiting growth. The combination of gallein and INH resulted in decreased levels of metabolites associated with several key metabolic pathways. It is plausible that the synergistic action of INH and gallein on *Mtb* growth is not solely due to the reduced integrity of the cell envelope, but also to the disruption of other metabolic pathways.

In conclusion, the combination of INH and gallein affects several aspects of *Mtb* physiology, and gallein thus potentiates INH antibiotic effects on *Mtb*. Because they are not present in mammalian cells (Brown and Kornberg, 2004), PPKs are attractive target for suppressing *Mtb* growth and elimination.

## Materials and methods

### Cell culture

Human peripheral blood was collected from healthy volunteers who gave written consent, and with specific approval from the Texas A&M University human subjects institutional review board. Peripheral blood mononuclear cells (PBMCs) were purified as previously described (Pilling et al., 2009). The PBMCs were cultured in RBCSG (RPMI-1640, # 15-040-CV, Corning, Corning, NY) containing 10% bovine calf serum [VWR Life Science Seradigm, Radnor, PA) and 2 mM L-glutamine (Lonza)], and where indicated containing 25 ng/mL human granulocyte-macrophage colony-stimulating factor (GM-CSF) ((# 572903, Biolegend, San Diego, CA) at 37°C in a humidified chamber with 5% CO_2_ in 96-well, tissue-culture-treated, polystyrene plates (type 353072, Corning) with 2 × 10^5^ cells in 200 μL in each well. At day 7, loosely adhered cells were removed by gentle pipetting and removing the medium, and fresh RBCSG containing GM-CSF (as described above) was added to the cells to a final volume of 200 μL per well, and *Mtb* survival assays were performed as described below.

The attenuated (mc-ΔleuDΔpanCD) Biosafety Level-2 strain of *Mtb*, which is a derivative of the H37Rv strain (Sampson et al., 2004), was a gift from Dr. Jim Sacchettini, Texas A&M University, College Station, TX and is referred to as *Mtb* in this paper. This strain was cultured according to the methods described in Larsen et al. (2007), Qi et al. (2013), and Rock et al. (2017) in Middlebrook 7H9 broth [Becton, Dickinson and Company (BD), Sparks, MD] containing 0.5% glycerol (VWR), 0.05% Tween 80 (MP Biomedicals, Solon, OH), Middlebrook Oleic ADC Enrichment (BD), 50 μg/mL leucine (VWR), and 50 μg/mL pantothenate (Beantown Chemical, Hudson, NH) either in a rotator (10 rpm) or on plates with 7H10 agar (BD) and the above additives. All cultures were incubated at 37°C in a humidified incubator. These supplemented cultures are hereafter referred to as 7H9S or 7H10S.

### *Mtb* growth assay

*Mtb* was grown in 24-well plates (type 353047, Corning). Each well was filled with 1 ml of 7H9S media, either containing no isoniazid (INH) or supplemented with 0.1, 1, 10, or 100 μg/ml INH (prepared from a 50 mg/ml stock in water, Cat#I3377, Sigma, Livonia, MI). *Mtb* samples obtained from log-phase liquid culture, as described above, were washed twice with 10 ml of 7H9S by centrifugation at 4,000 × g for 10 min. The optical density at 600 nm (OD_600_) was measured in a well of a 96-well, tissue-culture-treated, polystyrene plate (type 353072, Corning) at 600 nM with a Synergy Mx monochromator microplate reader (BioTek, Winooski, VT). The *Mtb* were then resuspended in an appropriate volume of 7H9S to achieve a final OD_600_ of 1. Ten microliters of *Mtb* with an OD_600_ of 1 were added to each well to reach a final OD_600_ of 0.01 in 1 ml. The plates were subsequently incubated in a container with humidity provided by wet paper towels at 37 °C in a humidified incubator. On the 21st day, the *Mtb* culture was gently resuspended, and 100 μl of the cells were transferred to a 96-well, tissue culture-treated plate (# 353072, Corning). The OD_600_ was measured using a microplate reader. Given that we observed complete inhibition of *Mtb* growth with 100 μg/ml INH in our experimental setup, we opted to utilize 1 μg/ml INH for all our assays. This choice aligns with previous reports from other research groups, which had documented the tolerance and resistance of *Mtb* to concentrations of INH >1 μg/ml (Ojha et al., 2008; Flentie et al., 2019).

To investigate the impact of gallein (3′,4′,5′,6′-Tetrahydroxyspiro[isobenzofuran-1(3H),9′-(9H)xanthen]-3-one) (Cat#3090, Tocris, Minneapolis, MN) and/or INH on *Mtb* growth, a *Mtb* culture plate was prepared as described method. However, in this case, each well of a type 353046, 6-well, tissue culture-treated plate (Corning) contained a final OD_600_ of 0.01 in 5 ml. *Mtb* was incubated with gallein at concentrations of 0.005, 0.05, 0.5, 5, or 50 μM and/or 1 μg/ml INH. A 50 mM gallein stock in DMSO (VWR) was diluted to 5 mM in 7H9S and further serially diluted in 7H9S to obtain lower concentrations. The control well contained 7H9S with DMSO, which was similarly serially diluted in 7H9S, as was done for gallein. The OD_600_ of the cells was measured daily for 14 days, and the *Mtb* growth curves were generated as a percentage of the OD_600_ on day 0.

To determine the viability of *Mtb* cells pre-exposed to INH and/or gallein for 14 days, 200 μl of cells from above experiments were washed twice with 1 ml of 7H9S, resuspended in 200 μl of 7H9S without INH and gallein 96-well plates (# 353072, Corning), and the OD_600_ of the cells was measured daily for 14 days, and the *Mtb* growth curves were generated as a percentage of the OD_600_ on day 0.

### Bacterial survival assay

To determine the effect of INH and/or Gallein on the survival of *Mtb* in human macrophages, human macrophages (from blood monocytes cultured with GM-CSF for 6 days were mixed with *Mtb* following (Rijal et al., 2020), in the absence or in the presence of 1 μg/ml INH and/or 5 μM Gallein. At day 7, after isolating monocytes from donor blood, after removing loosely adhered cells as described above, 200 μL RBCSGLP (RBCSG containing 50 μg/mL leucine and 50 μg/mL pantothenate) were added to macrophages in each well in 96-well, tissue-culture-treated, polystyrene plates (# 353072, Corning) and incubated for 30 min at 37°C. Meanwhile, 1 mL of *Mtb* from a log phase culture was washed twice with RBCSGLP without GM-CSF by centrifugation at 12,000 × g for 2 min in a microcentrifuge tube, resuspended in 1 mL of RBCSGLP, and the OD_600_ of 100 μl of the culture in a well in a 96-well plate (# 353072 Corning) was measured as above. Two-hundred microliter of RBCSGLP was used as a blank. The bacteria were diluted to an OD_600_ of 0.5 (~10^7^ *Mtb*/mL) in RBCSGLP. *Mtb* (~1 μl) was added to macrophages in each well such that there were ~5 bacteria per macrophage, considering ~20% of the blood monocytes converted to macrophages in the presence of GM-CSF (Cui et al., 2021). The bacteria-macrophage co-culture plate was centrifuged at 500 × g for 3 min with a Multifuge X1R Refrigerated Centrifuge (Thermo Scientific, Waltham, MA) to synchronize phagocytosis of the bacteria, and incubated for 2 h at 37°C. The supernatant medium was removed by gentle pipetting and was discarded. Two-hundred microliter of PBS warmed to 37°C was added to the co-culture in each well, cells were gently washed to remove un-ingested extracellular bacteria, the PBS was removed, and 200 μL of RBCSGLP with GMCSF in the absence or in the presence of 1 μg/ml INH and/or 5 μM gallein was added to the cells. After 2 h, cells were washed twice with PBS as above. Two-hundred microliter of RBCSGLP with GMCSF in the absence or in the presence of 1 μg/ml INH and/or 5 μM gallein was then added to the cells. After 4 and/or 48 h of infection, macrophages were washed as above with PBS, the PBS was removed, and cells were lysed using 100 μL 0.1% Triton X-100 (Alfa Aesar, Ward Hill, MA) in PBS for 5 min at room temperature by gentle pipetting, and 10 and 100 μL of the lysates were plated onto agar plates (as described above for *Mtb* culture). The *Mtb* containing agar plates were incubated for 3–4 weeks or until the *Mtb* colonies appeared. Bacterial colonies obtained from plating 10 and 100 μL lysates were manually counted, the number of viable ingested bacterial colonies per 10 and 100 μL lysates was calculated, and the number of viable ingested bacteria colony forming units (cfu) per ml of lysate was then calculated, which corresponds to the number of viable ingested bacteria in ~2 × 10^5^ macrophages. To calculate the percent of control, cfu/ml of the control was considered 100%.

### PolyP assays, RNA extraction, and quantitative reverse transcription PCR

Log phase *Mtb* cultures were prepared similarly to the growth assays in 6-well, tissue culture-treated plates (# 353046, Corning), with the exception that 5 μM gallein and/or 1 μg/ml INH were used. Plates were incubated in a container humidified with wet paper towels at 37°C for 1, 5, or 14 days. After incubation, 100 μl of cells were transferred to a 96-well, black/clear, tissue-culture-treated, glass-bottom plate (# 353219, Corning) for imaging as described below. The remaining cells were transferred to a 15-ml conical tube, harvested by centrifugation at 4,000 × g for 10 min, and 4.5 ml of the supernatant was transferred to a new 15-ml conical tube for extracellular polyP measurement. Cell pellets were further processed for RNA extraction, following the procedure outlined below. PolyP levels in the supernatant were assessed by adding 25 μg/ml of DAPI (Biolegend) (from a stock of 2 mg/ml) and measuring fluorescence at 415 nm excitation and 550 nm emission, as previously described (Aschar-Sobbi et al., 2008). PolyP standards (Sodium Polyphosphates, Glassy, Spectrum, New Brunswick, NJ) at concentrations of 0, 0.5, 1, 10, 100, 200, and 500 μg/ml were prepared in 7H9S. The polyP content was normalized to the total protein content, which was determined from the cell lysates as described below.

For RNA extraction, cell pellets obtained from the treatments described above were resuspended in 250 μL of GITC lysis buffer (containing 4 M guanidine isothiocyanate and 50 mM Tris-HCl at pH 7). The cells were lysed by incubating at 95°C for 10 min. Ten microliters of the lysates were used to determine the total protein content using a Bradford assay. The remaining lysates were used to extract RNA using an RNA extraction kit (Zymo Research, Irvine, CA). Complementary DNA (cDNA) was synthesized from 2 μg of RNA using the Maxima H Minus First Strand cDNA Synthesis kit (Thermo Scientific). Quantitative PCR was performed using SYBR GreenER™ qPCR SuperMix Universal reagent (Thermo Scientific), following the manufacturer's instructions using a QuantStudio (TM) 6 Flex thermal cycler (Thermo Scientific). The levels of *Mtb's ppk1* and *ppk2* mRNAs were determined using the gene-specific primers listed in Supplementary Table S2.

### Fluorescence microscopy

To determine the localization of polyP in *Mtb*, 100 μl of *Mtb* cells from the growth assay on Day 21 or the growth assay on Days 1, 5, or 14 were transferred to a 96-well, black/clear, tissue-culture-treated, glass-bottom plate (# 353219, Corning) or cells smears were prepared on Superfrost micro glass slides (Cat#48311-703, VWR) as described previously (Pilling et al., 1989). The cells were fixed with 4% (wt/vol) paraformaldehyde (Cat#19210, Electron Microscopy Sciences, Hatfield, PA) in PBS for 10 min. After fixation, the cells were washed two times with 300 μl of PBS, blocked with 1 mg/ml BSA (Thermo Scientific) in PBS, and then stained with 10 μg/ml of GFP-PPX (provided generously by Dr. Ursula Jacob from the University of Michigan) in PBS/0.1% Tween 20 (PBST; Fisher Scientific) (Xie et al., 2019). Following staining, the *Mtb* cells were washed three times with PBST, and 200 μl of PBS was added to the well, or coverslips were mounted on slides with smears with Vectashield hard set mounting medium (Cat# H-1800, Vector Labs, Burlingame, CA) and left to dry overnight in darkness. Images of *Mtb* were captured using a 100 × oil-immersion objective on a Nikon Eclipse Ti2 (Nikon, Kyoto, Japan), and image deconvolution was performed using the Richardson–Lucy algorithm (Laasmaa et al., 2011) in NIS-Elements AR software (Nikon). The integrated fluorescence density was measured in randomly selected individual cells manually using the freehand selection feature in Fiji (ImageJ) (Schindelin et al., 2012).

### Transmission electron microscopy

*Mtb* cells treated with 5 μM gallein and/or 1 μg/ml INH for 14 days were prepared as described for the growth assays above. A volume of 100 μl of cells in 7H9S was fixed by adding an equal volume of 2 × fixative, which contained 84 mM NaH_2_PO_4_, 68 mM NaOH, 4% paraformaldehyde (Cat#19210, Electron Microscopy Sciences), and 1% glutaraldehyde (Cat#0875, VWR). The samples were gently rocked for 1 h and then stored at 4°C. Sample preparation for TEM imaging was performed by the Texas A&M University Microscopy and Imaging Center Core Facility's staff (RRID: SCR_022128). Briefly, on the following day, the fixed samples were collected by centrifugation for 5 min at 14,000 × g. Subsequently, they were postfixed and stained for 2 h with 1% osmium tetroxide in 0.05 M HEPES at pH 7.4. The samples were then collected by centrifugation and washed with water five times, and dehydrated with acetone according to the following protocol: 15 min in 30, 50, 70, and 90% acetone each, followed by three changes of 100% acetone, each lasting 30 min. During the final wash step, a minimal amount of acetone was retained, just enough to cover the pellets, to prevent rehydration of the samples. Subsequently, the samples were infiltrated with modified Spurr's resin (Quetol ERL 4221 resin; Electron Microscopy Sciences; RT 14300) in a Pelco Biowave processor (Ted Pella, Inc., Redding, CA). The process included 1:1 acetone-resin for 10 min at 200 W (no vacuum), 1:1 acetone-resin for 5 min at 200 W (vacuum at 20 inches Hg, with vacuum cycles involving open sample container caps), and 1:2 acetone-resin for 5 min at 200 W (vacuum at 20 inches Hg). This was followed by four cycles of 100% resin for 5 min each at 200 W (vacuum at 20 inches Hg). The resin was then removed, and the sample fragments were transferred to BEEM conical-tip capsules that were prefilled with a small amount of fresh resin. More resin was added to fill the capsules, and they were left to stand upright for 30 min to ensure that the samples sank to the bottom. The samples were polymerized at 65°C for 48 h in an oven and then left at room temperature (RT) for an additional 24 h before sectioning. Sections of 70–80 nm thickness were obtained using a Leica UC/FC7 ultramicrotome (Leica Microsystems), deposited onto 300-mesh copper grids, and stained with uranyl acetate-lead citrate. Grids were imaged using a JEOL 1200 EX TEM operating at 100 kV. To avoid discrepancies, the cell envelope thickness of distinct cells found in each grid was measured using ImageJ at random places on the envelope. Cells with no intact cell envelope were scored as having a thickness of 0 nm. Cells in clumps were not scored for cell envelope thickness. Values determined for individual cells were plotted.

### Metabolic activity assay

Cell proliferation and viability in *Mtb* can be assessed by incubating the cells with resazurin reagent for 6–12 h, and monitoring the color change from blue to purple (Franzblau et al., 1998). Metabolically active cells transform the non-fluorescent blue dye (resazurin) into a fluorescent pink product (resorufin), while inactive cells rapidly lose their metabolic capacity and, consequently, do not generate a fluorescent signal (Lescat et al., 2019). *Mtb* from the log phase culture were prepared as previously described for the proliferation assay, with 100 μl of cells being prepared per well in 96-well, tissue culture-treated plate (# 353072, Corning). *Mtb* was treated with 5 μM gallein and/or 1 μg/ml INH, and the plate containing the *Mtb* was incubated at 37 °C for 24 h. Cells were then incubated with prewarmed Deep Blue Cell Viability resazurin dye (Biolegend) to a final concentration of 10% in each well for 12 h (Franzblau et al., 1998). The fluorescence signal was then measured using a microplate reader following the manufacturer's protocol.

### Metabolomics

*Mtb* cells from log-phase cultures were prepared as described for the growth assays, with the exception that 10 ml of culture was prepared. The *Mtb* cells were treated with 5 μM gallein and/or 1 μg/ml INH. After 24 h, 10 ml of the culture was collected by centrifugation at 4,000 × g for 10 min at 4°C. The cells were then washed twice with 10 ml of chilled (0–4°C) phosphate-buffered saline (PBS) to prevent metabolite contamination from the culture media. The cells were resuspended in 10 ml of PBS (Mackay et al., 2015), and 100 μl of this suspension was transferred to a 96-well plate to measure the OD_600_. The remaining cell suspension was centrifuged again at 4,000 × g for 10 min at 4°C to collect the cell pellets for metabolite extraction. After the final wash, excess PBS was carefully removed from all the samples. The day before the assay, 10 ml of extraction solvent [acetonitrile (BDH83639.400, VWR): methanol (BDH20864.400, VWR):water (40:40:20)] was prepared and stored at −70°C. On the day of the experiment, heavy amino acid standards (Metabolomics amino acid mix standard, Cat# MSK-A2-1.2, Cambridge Isotope Laboratories, Tewksbury, MA) were added as a spike to the extraction solvent to 5 μM final concentration. To halt bacterial metabolism, the *Mtb* pellets were immediately suspended in 200 μl of spiked extraction solvent that had been pre-cooled on dry ice (Nandakumar et al., 2014). *Mtb* disruption was achieved using a Mini-beadbeater-16 (BioSpec Products, Bartlesville, OK). The *Mtb* samples (200 μl) were placed in 2 ml Polypropylene Microvials (Cat#10832, BioSpec Products) containing approximately 70 μl of 0.1 mm Zirconia/Silica beads (Cat# 11079101z, BioSpec Products). Bead-beating was performed in three cycles of 1 min at 3,450 oscillations/min, with 2 min of cooling on ice between cycles (Mehra and Philips, 2014). Subsequently, the microvials containing the disrupted cells and zirconium beads were incubated at −20°C for 20 min. They were then clarified by centrifugation at 8,000 × g for 15 min at 4 degrees Celsius. The resulting supernatant was filtered through a 0.22 μm filter (Cat# UFC30GV25, Merck Millipore, Cork, IRL) into 0.2 ml glass Stepvial inserts (Cat# 200 238, ThermoScientific). These step vials were placed inside 0.25 ml polypropylene vials with polypropylene caps with PTFE/silicone septa (Cat# 200 410, ThermoScientific). The pellets containing beads were resuspended in 100 μl of Radioimmunoprecipitation assay buffer (RIPA) (Cat#89900, Thermo Scientific) containing 1X protease and phosphatase inhibitor cocktail (Cat#1861281, Thermo Scientific), incubated on ice for 15 min, clarified by centrifugation at 10,000 × g for 5 min at 4°C, and 25 μl of the supernatant was used to determine the protein amount following the manufacturer's instructions using a Pierce BCA Protein Assay Kit (Cat#23225, Thermo Scientific). A vial containing a quality control mixture pool of 12.5 μl from each sample (150 μl total) was prepared along with the other samples. These vials were sealed with Parafilm and stored at −80°C before the untargeted metabolomics analysis (Mackay et al., 2015). Samples were analyzed with a Shimadzu high-performance liquid chromatography (HPLC) (Nexera X2 LC-30AD, Kyoto, Japan) coupled to a Sciex TripleTOF 6600 high-resolution mass spectrometer (HRMS) for the separation and detection of various classes of metabolites in the samples using an untargeted metabolomics approach at the UTSW metabolomics core facility (https://www.utsouthwestern.edu/research/core-facilities/metabolomics/). Metabolites detected in at least three biological replicates were considered for further analysis. The peak area of metabolites was normalized to the total protein content determined from *Mtb* lysates, and pathway enrichment analysis was conducted using the online analytical tool Metaboanalyst (www.metaboanalyst.ca) and the BioCyc Database, employing the *Mtb* H37Rv reference genome (Karp et al., 2019).

### Statistical analysis

Statistical analyses were performed using Prism 10 (GraphPad Software, Boston, MA) or Microsoft Excel. *P* < 0.05 was considered significant.

## Conclusions

*Mycobacterium tuberculosis* (*Mtb*) is the causative agent of tuberculosis (TB), which is responsible for more deaths than any other infectious disease. The alarming prevalence of drug-tolerant *Mtb* strains has further exacerbated this global health crisis. Some pathogenic bacteria such as *Mtb* appear to increase levels of polyphosphate as a defense against antibiotics. We found that gallein, a small molecule inhibitor of bacterial polyphosphate kinases, strongly potentiates the ability of the frontline anti-tuberculosis drug isoniazid (INH) to inhibit the growth of *Mtb* both alone and in human macrophages. This has unveiled vulnerabilities in *Mtb* that could be strategically leveraged to reverse INH tolerance.

## Data availability statement

The original contributions presented in the study are included in the article/Supplementary material, further inquiries can be directed to the corresponding authors.

## Ethics statement

The studies involving humans were approved by Texas A&M University human subjects Institutional Review Board. The studies were conducted in accordance with the local legislation and institutional requirements. The participants provided their written informed consent to participate in this study.

## Author contributions

RR: Conceptualization, Formal analysis, Investigation, Validation, Writing—original draft, Writing—review & editing. RG: Conceptualization, Funding acquisition, Supervision, Validation, Writing—review & editing.
