# Supplementary material for: Gallein potentiates isoniazid's ability to suppress Mycobacterium tuberculosis growth
Source: Front Microbiol. 2024 Apr 15;15:1369763. doi: 10.3389/fmicb.2024.1369763 (PMC11060752; doi:10.3389/fmicb.2024.1369763)
Supplement: Supplementary file 1 [file Data_Sheet_1.docx]

**
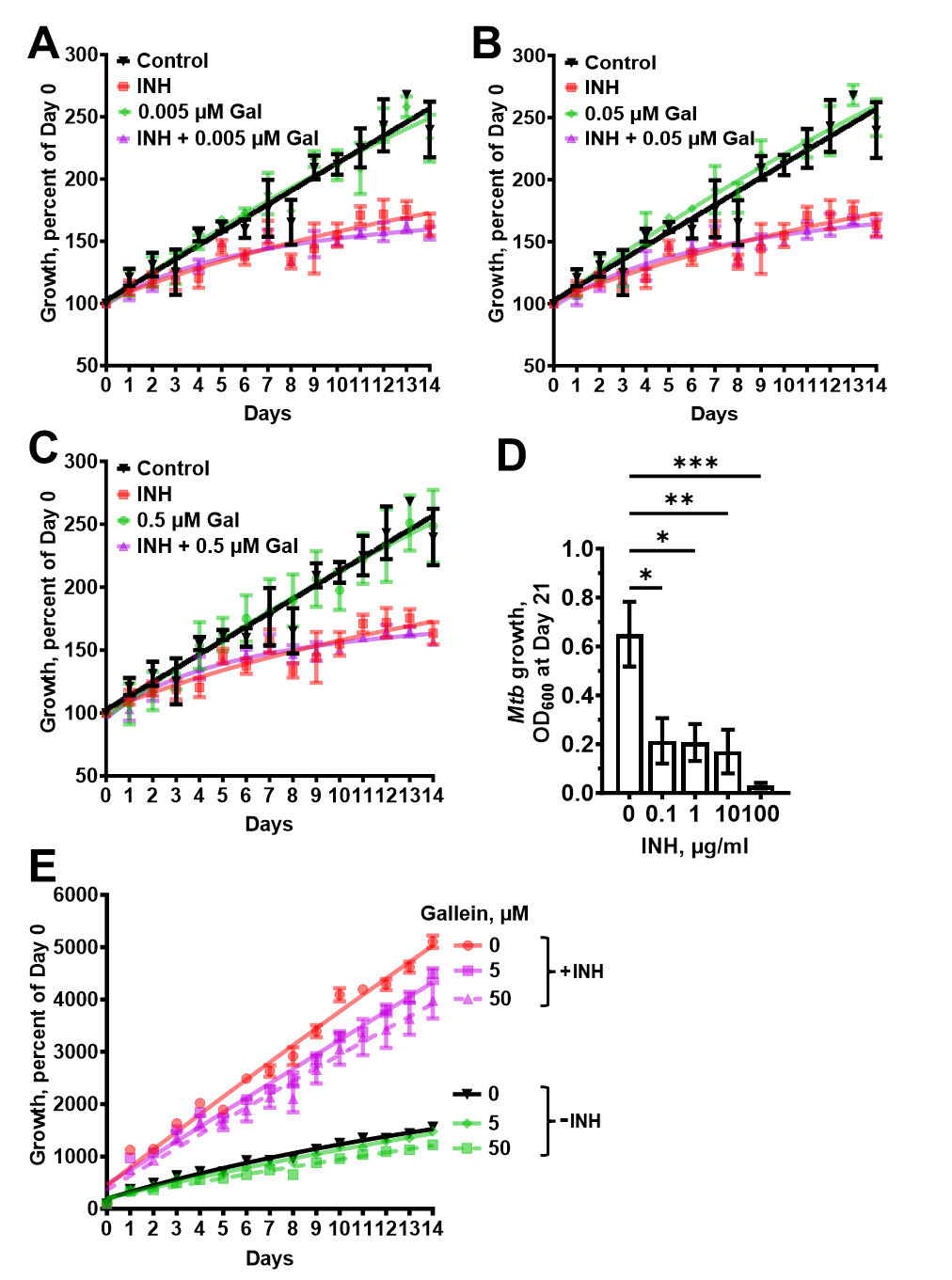
*Supplementary Material***

**Supplementary Figure 1**: INH reduces *Mtb* growth in *in vitro* culture. (A - C) *Mtb* cultures were grown for 14 days in the absence (Control) or presence of 1 µg/ml INH and/or 0.005 µM (A), 0.05 µM (B), or 0.5 µM (C) gallein. The OD_600_ was measured daily, and growth was determined as a percentage of Day 0 OD_600_. (D) *Mtb* were cultured for 21 days in the presence of the indicated concentrations of INH, and the OD_600_ was measured on Day 21. (E) *Mtb* at day 14 from Figures 1A and 1B were washed, regrown in the absence of INH or gallein for 14 days, and the OD_600_ was measured daily. Growth was determined as a percentage of the day 0 OD_600_. All values are mean ± SEM of three independent experiments. * P < 0.05; ** P < 0.01; *** P < 0.001 (One-way ANOVA with Dunnett's multiple comparisons test).


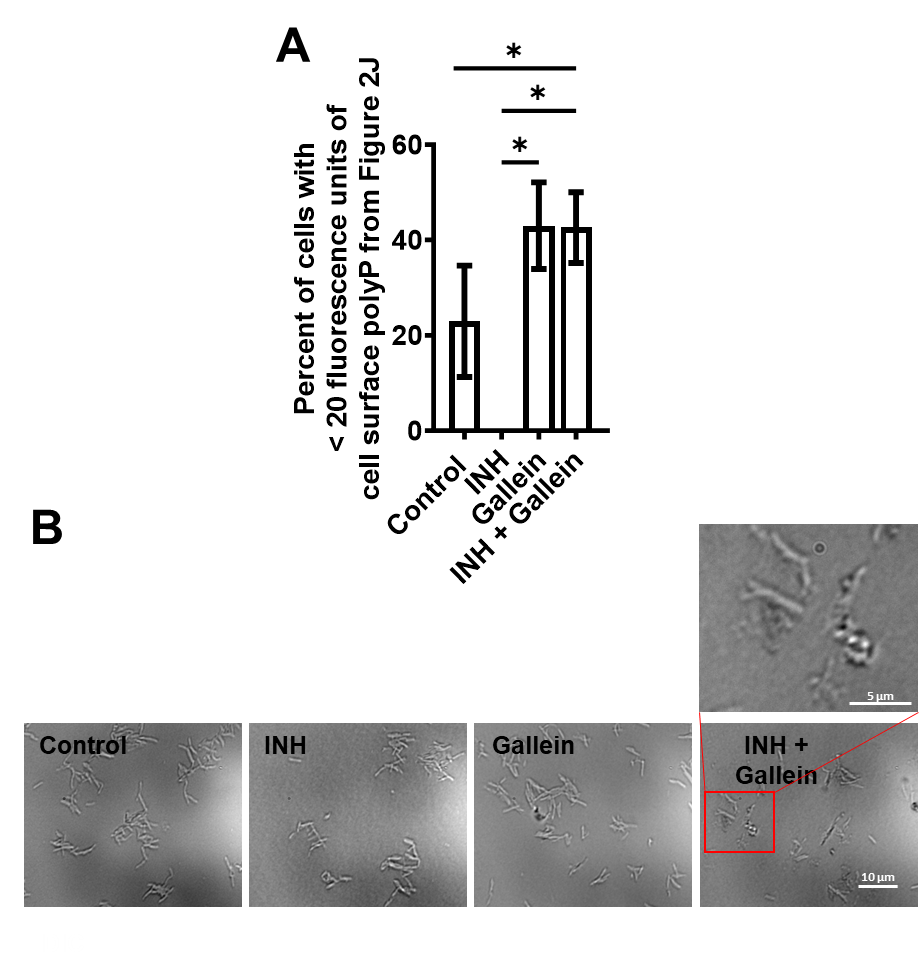
**Supplementary Figure 2**: INH and gallein increase percentages of *Mtb* cells with reduced cell surface polyP and cellular debris accumulation. (A) The percentages of cells with cell surface polyP levels less than 20 fluorescence units from Figure 2J. All values are mean ± SEM of three independent experiments. * P < 0.05 (One-way ANOVA with Tukey's multiple comparisons test, and t-tests between Control and the combination of INH and Gallein. (B) Differential interference contrast (DIC) images of *Mtb* from Figure 2J. Representative images from at least three independent experiments are shown. Bars are 10 µm for main images and 5 µm for inset. White arrows indicate cellular debris.

**
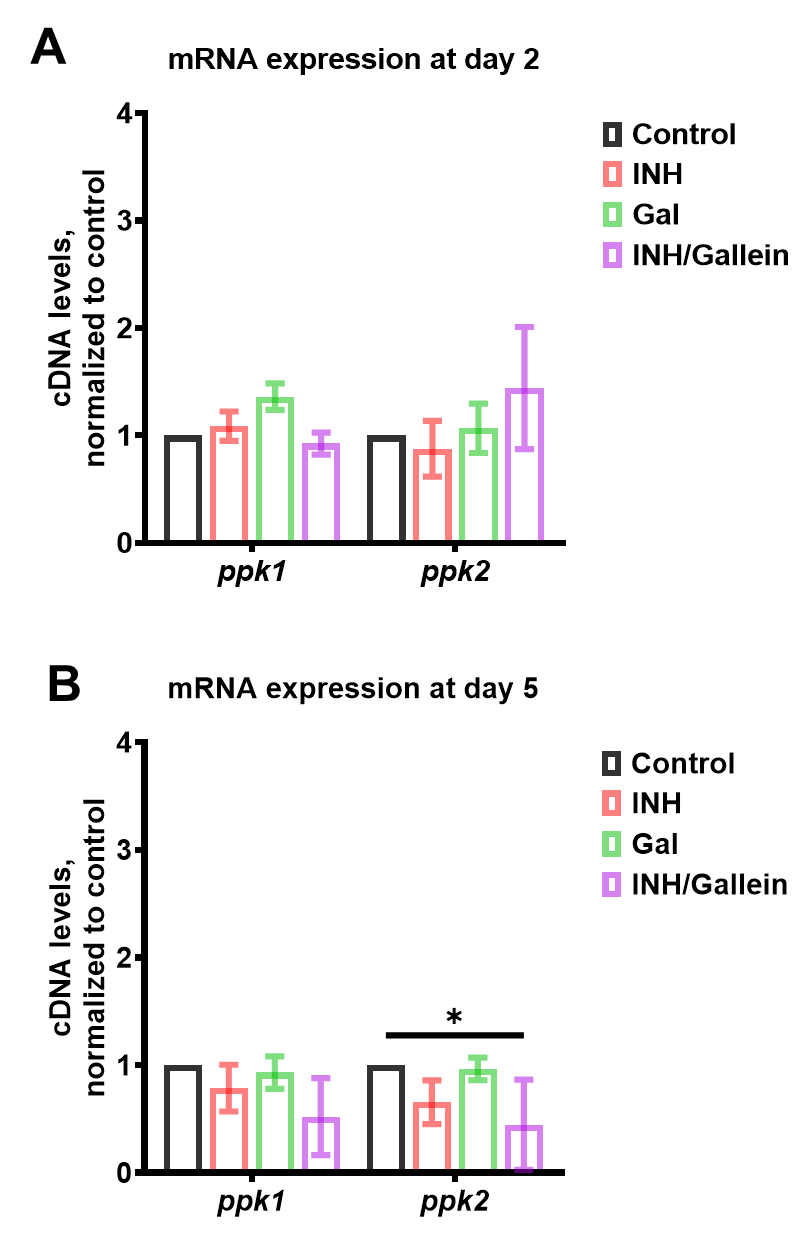
Supplementary Figure 3**: INH or gallein does not alter *ppk1* and *ppk2* mRNA levels. Total RNA was extracted from *Mtb* cultures grown for 1 day (A) or 5 days (B) in the absence or presence of 1 µg/ml isoniazid (INH) and/or 5 µM gallein. The RNA was reverse-transcribed to generate cDNA, and the levels of *ppk1* and *ppk2* cDNA were quantified by quantitative PCR using gene-specific primers (Supplemental Table 2). The cDNA level from untreated *Mtb* (Control) was set to 1. All values are mean ± SEM of three independent experiments.


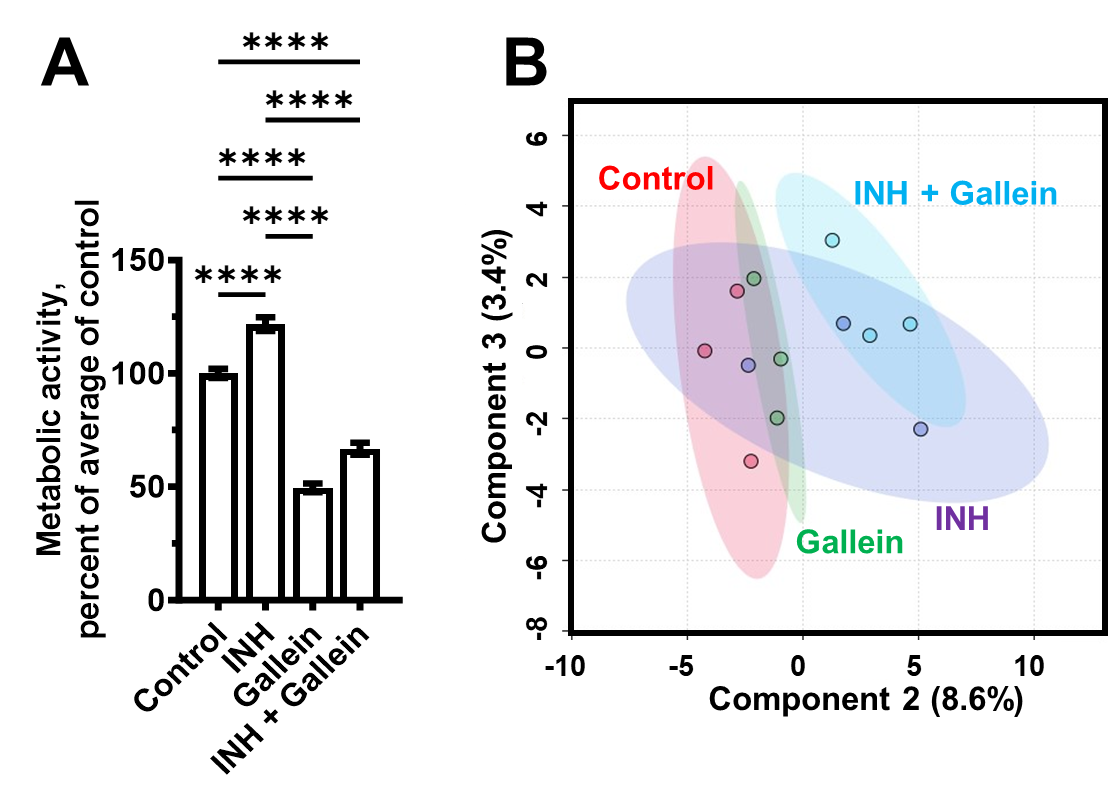
**Supplementary Figure 4**: Gallein inhibits *Mtb* metabolic activity. (A) *Mtb*, treated without or with 1 µg/ml INH and/or 5 µM gallein for 24 hours, were incubated with cell viability dye for 12 hours, and fluorescence was measured. The average of the control was considered 100%. (B) The score plot of the two principal components (2 and 3) of a principal component analysis model, built on the entire metabolomics dataset (Figure 4) from three independent experiments, is color-coded by group with confidence ellipses. Control datasets are red circles, INH-treated datasets are purple circles, gallein-treated datasets are green circles, and INH and gallein-treated datasets are blue circles. The contribution ratios (variance) of the two principal components are shown in parentheses. All values represent the mean ± SEM of three independent experiments for (A). **** P < 0.001 (One-way ANOVA with Tukey's multiple comparisons test).

**Supplementary Table 1**: Gallein potentiates the ability of INH to inhibit Mtb growth within macrophages. Viable ingested Mtb in macrophages, in the absence (Control) or presence of 1 µg/ml INH and/or 5 µM gallein, was determined as colony-forming units (CFU) at 4 hours and 48 hours after ingestion. All values are mean ± SD of four (2 females and 2 males) independent experiments.

|  | **CFUs at 4 h (Mean ± SD)** | **CFUs at 48 h (Mean ± SD)** |
| --- | --- | --- |
| Control | 444 ± 354 | 712 ± 809 |
| 1 µg/ml INH | 403 ± 645 | 22 ± 5 |
| 5 µM gallein | 333 ± 584 | 170 ± 70 |
| 1 µg/ml INH + 5 µM gallein | 58 ± 101 | 0 ± 0 |

**Supplementary Table 2**: Oligonucleotides for quantitative real-time polymerase chain reaction (qPCR).

| **cDNA** | **5’-3’ Forward Primer** | **5’-3’ Reverse Primer** |
| --- | --- | --- |
| ***ppk1­*** | TCGAGCACCACGCATTT | ATCTGCCACAACGACGATAG |
| ***ppk2*** | AATAGTCCTCCCACCGATACA | CCAAAGGTGAGATAGTGCTCTT |
